# Measurement of the Applicability of Abdominal Point-of-Care Ultrasound to the Practice of Medicine in Saudi Arabia and the Current Skill Gaps

**DOI:** 10.24908/pocus.v6i1.14761

**Published:** 2021-04-22

**Authors:** Rajkumar Rajendram, Mamdouh Souleymane, Naveed Mahmood, Rakan Sambas, Yousuf M S Kharal

**Affiliations:** 1 Department of Medicine, King Abdulaziz Medical City, King Abdulaziz International Medical Research Center, Ministry of National Guard - Health Affairs Riyadh Saudi Arabia; 2 College of Medicine, King Saud bin Abdulaziz University of Health Sciences Riyadh Saudi Arabia; 3 College of Medicine, Alfaisal University Riyadh Saudi Arabia

**Keywords:** Abdominal point-of-care ultrasound, Hepatomegaly, Splenomegaly, Ascites, Hydronephrosis, Education needs assessment, Curriculum development, Residency training, Fellowship training

## Abstract

**Background:** Renal, gastrointestinal, and hepatic pathology, and the resources available for their management vary internationally. Whilst abdominal point-of-care ultrasound (APOCUS) should enhance management, uptake by physicians, worldwide, has been poor. So, the aim of this study was toexplore the applicability of APOCUS to medical practice in Saudi Arabia, residents’ current ability to perform APOCUS, and the skill gaps. **Methods: **A validated questionnaire was distributed to theinternal medicine residents at our institution to determine their ability to perform APOCUS (self-reported), and obtain their opinions on its applicability for the detection of hepatomegaly, splenomegaly, hydronephrosis, and ascites. **Statistical analysis:** Standard descriptive statistical techniques were used. Categorical data, presented as frequency, were compared using the χ^2^ test. The Likert scale responses, presented as mean ± standard deviation, were compared with a t test or analysis of variance. **Results: **Ninety-eight residents participated (response rate 90.7%). Abdominal POCUS is very applicable to their practice. The use of APOCUS to detect ascites was the most applicable (mean 4.61 ± SD 0.69). However, proficiency in APOCUS was poor (mean 1.65 ± SD 1.11). **Conclusions:** The difference between internists’ self-reported ability to perform APOCUS and its perceived usefulness demonstrates a skill gap. Thus, whilst APOCUS is applicable to medical practice in Saudi Arabia, significant skill gaps exist.

## Introduction

Diagnostic point of care ultrasound (POCUS) of the abdomen (APOCUS) is a fast, portable, non-invasive, diagnostic tool 1, 2, 3, 4. The diagnostic accuracy of APOCUS for the detection of many intra-abdominal pathologies (e.g., hepatomegaly 3, splenomegaly 4, hydronephrosis 1, 2, and ascites 3) is excellent. Screening for hepatomegaly, splenomegaly, and ascites is amongst the commonest indications for APOCUS 3, 4, and the management of a patient with abdominal distension can be expedited if the treating physician uses APOCUS to answer the question: "does the patient have cirrhosis and portal hypertension leading to ascites?" 

Thus, APOCUS is an invaluable adjunct to bedside diagnostic evaluation 1, 2, 3, 4, 5, 6, that should be of great value to internists, nephrologists, gastroenterologists, and hepatologists. However, practicing physicians have been reluctant to integrate this paradigm-shifting technology into their routine practice 1.

It is argued that the availability of radiology services reduces the need for other physicians to perform imaging. However, this is in part because besides radiologists, most physicians lack of familiarity with the use of ultrasound 1. As APOCUS is relatively new technology, most internists, nephrologists, gastroenterologists, and hepatologists have little or no experience of its use. Furthermore, POCUS is highly operator dependent 7. To be effective, APOCUS must be performed by competent practitioners 1, 6. 

Despite these limitations, there is increasing interest among internal medicine (IM) residents for additional training in ultrasound 8. Safe, competent, and effective use of POCUS requires training to close gaps in learners’ knowledge and skills 9, 10. Fortunately, recent data suggests that those keen to learn APOCUS can obtain adequate proficiency with minimal training 3, 11. However, the integration of APOCUS into routine clinical practice still requires significant initial investment to cover the financial costs and train providers.

Many countries have developed APOCUS curricula for their internists 12, 13, 14, 15, and nephrologists 6. However, Saudi Arabia does not, as yet, have a syllabus for training IM residents or fellows in nephrology, gastroenterology or hepatology in APOCUS. As the Middle Eastern spectra of renal, gastrointestinal and hepatic pathologies differs from that in other regions 16, Western clinical practice may not be applicable to Saudi Arabia. 

The high initial investment required to develop an APOCUS service must be justified. Thus, to confirm that APOCUS is applicable to the current practice of medicine in Saudi Arabia, an assessment of needs is required 9, 17.

The aim of this study was to determine IM residents’ perceptions on the applicability of APOCUS, and by quantifying their self-reported ability to perform APOCUS; define the skill gaps in tertiary healthcare in Saudi Arabia.

## Subjects and Methods

### Ethical approval

Ethical approval for the protocol for this study (RC19/213/R) was obtained from the institutional review board (IRB) of the King Abdullah International Medical Research Center, Riyadh, Saudi Arabia. 

### Study design

This cross-sectional survey of IM residents was performed in King Abdulaziz Medical City, Riyadh; an academic, tertiary referral centre in Saudi Arabia. Procedures followed were in accordance with the ethical standards of the responsible institutional committee on human experimentation and with the Helsinki Declaration of 1975, as revised in 2000.

### Survey development

Studies describing the applications of APOCUS and the competencies required for its safe practice by internists, nephrologists, hepatologists and gastroenterologists were reviewed 1, 2, 3, 4, 5, 6, 9, 12, 18, 19. Two researchers with expertise in IM, APOCUS, and survey design (MS, and RR) used this literature data to develop a validated questionnaire to investigate the applicability of APOCUS to physicians. The questionnaire had three sections. The first section requested demographic data (i.e. gender, postgraduate year of training) The second section included questions on the applicability of four diagnostic applications of APOCUS (i.e. a needs assessment). The applicability of using APOCUS to detect hepatomegaly, splenomegaly, hydronephrosis, and abdominal free fluid was investigated. For each diagnostic application, participants were asked: How applicable is this indication for APOCUS to your practice? The third section asked participants to describe their ability to perform APOCUS (i.e. knowledge of APOCUS and proficiency in the interpretation of APOCUS findings). This section included a single self-reported question on their knowledge of APOCUS and their proficiency in the interpretation of APOCUS findings. This question was included to provide a summative overview of the respondents’ self-assessed ability to perform APOCUS.

After ethical approval, the survey (Appendix 1) was then pilot tested with three paediatric residents to obtain input on survey length, content, and clarity. It was universally agreed that no changes were required.

### Participants

During the academic year 01/10/18 – 30/09/19 there were 108 IM residents (postgraduate year [PGY] 1–4). Assuming a response distribution of 50%, it was estimated that 85 residents would be required to participate to obtain a 5% margin of error at a level of confidence of 95%. All IM residents at our institution were invited to participate. The final paper questionnaire was distributed to IM residents in August 2019. No incentives were provided. Written informed consent was obtained before participation in the survey. 

### Study outcomes

A 5-point Likert scale (1 very poor, 2 poor, 3 fair, 4 good, 5 very good) was used to assess the perceived applicability of four indications for APOCUS in the practice of IM in Saudi Arabia. The same 5-point Likert scale was used to assess self-reported ability to perform APOCUS. The skill gap in APOCUS was determined from the difference between residents’ perception of the applicability of APOCUS to their practice and their self-reported ability to perform APOCUS.

### Statistical analysis

The data were analysed using standard descriptive statistical techniques. The final analysis included all responses. The Cronbach’s alpha coefficient was used to determine the internal consistency of the subgroups of questions measuring applicability in the questionnaire. Residents’ responses were stratified by PGY. To facilitate the comparison of data, interval data, described on a 5-point Likert scale, were presented as both frequency and mean ± SD, as described previously.^9^ The data were compared using Student’s t-tests or analysis of variance (ANOVA) as appropriate. Categorical variables were compared using a Chi-squared test. All analyses were performed using Excel version 2016 (Microsoft, USA).

## Results

### Demographic data and response rates

The participants’ demographic details and response rates are shown in Table 1. The response rate (RR) was very high (90.7%) and exceeded that required to achieve the desired margin of error and level of confidence. Ninety-eight (male 73; female 25) of 108 (male 77; female 31) IM residents participated in our study. Although, female IM participants’ RR (80.6%) was significantly lower than that of the men (94.8%; χ^2^ 5.27, P=0.022); there were no statistically significant differences between the responses of male and female IM residents.

**Table 1:** Demographic data and response rates. The table presents the sample’s demographics and response rates. Response rates are stratified by postgraduate year (PGY) of training. Data are presented as frequency and percentage of strata totals. N, number of responses.

**Grade**	**N (RR % PGY)**
	**Total**
**PGY 1**	31 (93.9%)
**PGY 2**	25 (89.3%)
**PGY 3**	23 (82.1%)
**PGY 4**	19 (100%)
**Total**	98 (90.7%)

### Applicability of APOCUS to IM practice in Saudi Arabia

The applicability of the four indications for diagnostic APOCUS to IM practice in Saudi Arabia are shown in Tables 2 and 3. Cronbach’s alpha was 0.79 suggesting that the internal consistency of the responses of these questions was good. There were no statistically significant differences between the groups’ means as determined by one-way ANOVA (F(3,388)=1.79, P=0.15). The combined applicability of all indications of APOCUS was very high (mean applicability 4.46 ± SD 0.85; 379 responses (96.7%) were fair, good or very good; 342 responses (87.2%) were good or very good). 

Scanning to detect abdominal free fluid was the most applicable (mean applicability 4.61 ± SD 0.69). The participants considered scanning for hydronephrosis (mean applicability 4.33 ± SD 1.04), hepatomegaly (mean applicability 4.41 ± SD 0.83), and splenomegaly (mean applicability 4.42 ± SD 0.82) to be slightly less relevant.

**Table 2:** Residents’ perception of the applicability of APOCUS and their ability to perform APOCUS. This table presents residents’ perception of the applicability of APOCUS to their clinical practice and their self-reported ability to perform APOCUS. Applicability and proficiency are rated on a 5-point Likert Scale (1, Very Poor; 2, Poor; 3, Fair; 4, Good and 5, Very Good). Data are stratified by postgraduate year of training (PGY) and presented as mean ± standard deviation.

**Grade/Gender**	**Application of Abdominal Point of Care Ultrasound** **(Mean ± SD)**				**Ability** **(Mean ± SD)**
	**Hydronephrosis**	**Hepatomegaly**	**Splenomegaly**	**Ascites**	**APOCUS**
**PGY 1**	4.4 ± 1.2	4.4 ± 0.9	4.5 ± 0.9	4.6 ± 0.8	1.8 ± 1.2
**PGY 2**	4.2 ± 1.2	4.2 ± 0.9	4.3 ± 0.9	4.6 ± 0.6	1.6 ± 1.1
**PGY 3**	4.5 ± 0.7	4.6 ± 0.6	4.5 ± 0.7	4.6 ± 0.7	1.7 ± 1.1
**PGY 4**	4.4 ± 0.9	4.5 ± 0.8	4.5 ± 0.8	4.6 ± 0.6	1.6 ± 0.9
**Overall**	4.3 ± 1.0	4.4 ± 0.8	4.4 ± 0.8	4.6 ± 0.7	1.7 ± 1.1

**Table 3:** Residents’ responses to questions on the applicability of APOCUS to their clinical practice and self-reported ability to perform APOCUS. This table presents residents’ responses to questions on the applicability of four indications for abdominal point of care ultrasound (APOCUS) and self-reported ability to perform APOCUS. Applicability and proficiency are rated on a 5-point Likert Scale (1, Very Poor; 2, Poor; 3, Fair; 4, Good and 5, Very Good). Data are presented as frequency.

**Response** **(Likert scale)**	**Application of Abdominal Point of Care Ultrasound**				**Ability**
	**Hydronephrosis**	**Hepatomegaly**	**Splenomegaly**	**Ascites**	**APOCUS**
**Very Poor**	4	1	1	1	66
**Poor**	3	2	1	0	12
**Fair**	9	10	12	6	10
**Good**	22	27	25	22	7
**Very Good**	60	58	59	69	3
**Total**	98	98	98	98	98

### IM residents’ ability to perform APOCUS and assessment for skill gaps

The self-reported ability to perform APOCUS is displayed in Tables 2 and 3. The IM residents generally reported poor ability to perform APOCUS (mean 1.66 ± SD 1.11). When stratified by PGY (Table 2), no differences between residents’ abilities to perform APOCUS were identified (i.e. there were no statistically significant differences between the groups’ means as determined by one-way ANOVA (F(3,94)=0.18, P=0.91)). Thus, junior and senior residents’ self-reported abilities were similar and poor.

The self-reported ability to perform APOCUS was significantly lower than the IM residents’ perception of the applicability of APOCUS for detection of hydronephrosis (i.e. the indication for APOCUS perceived to be least useful; mean 4.33 ± SD 1.04; P < 0.0001), suggesting the presence of a skill gap. The skill gaps did not differ between junior and senior residents (i.e. there were no statistically significant differences between the PGY groups’ means as determined by one-way ANOVA (F(3,94)=0.11, P=0.95)). 

## Discussion

Abdominal POCUS is an accurate tool for investigating abdominal disease.^1^, ^2^, ^3^, ^4^ However, the spectra of abdominal diseases and facilities available within the Middle East varies significantly from that in other regions 16.

To justify the high investment required to develop an APOCUS training program, it is important to confirm that IM residents in Saudi Arabia require this skill. The current study therefore describes IM residents’ perception of the applicability of four indications for APOCUS to their practice at a medical city in Saudi Arabia.

### Residents’ perception of the applicability of APOCUS and their self-reported ability to perform APOCUS

**Figure 1:**
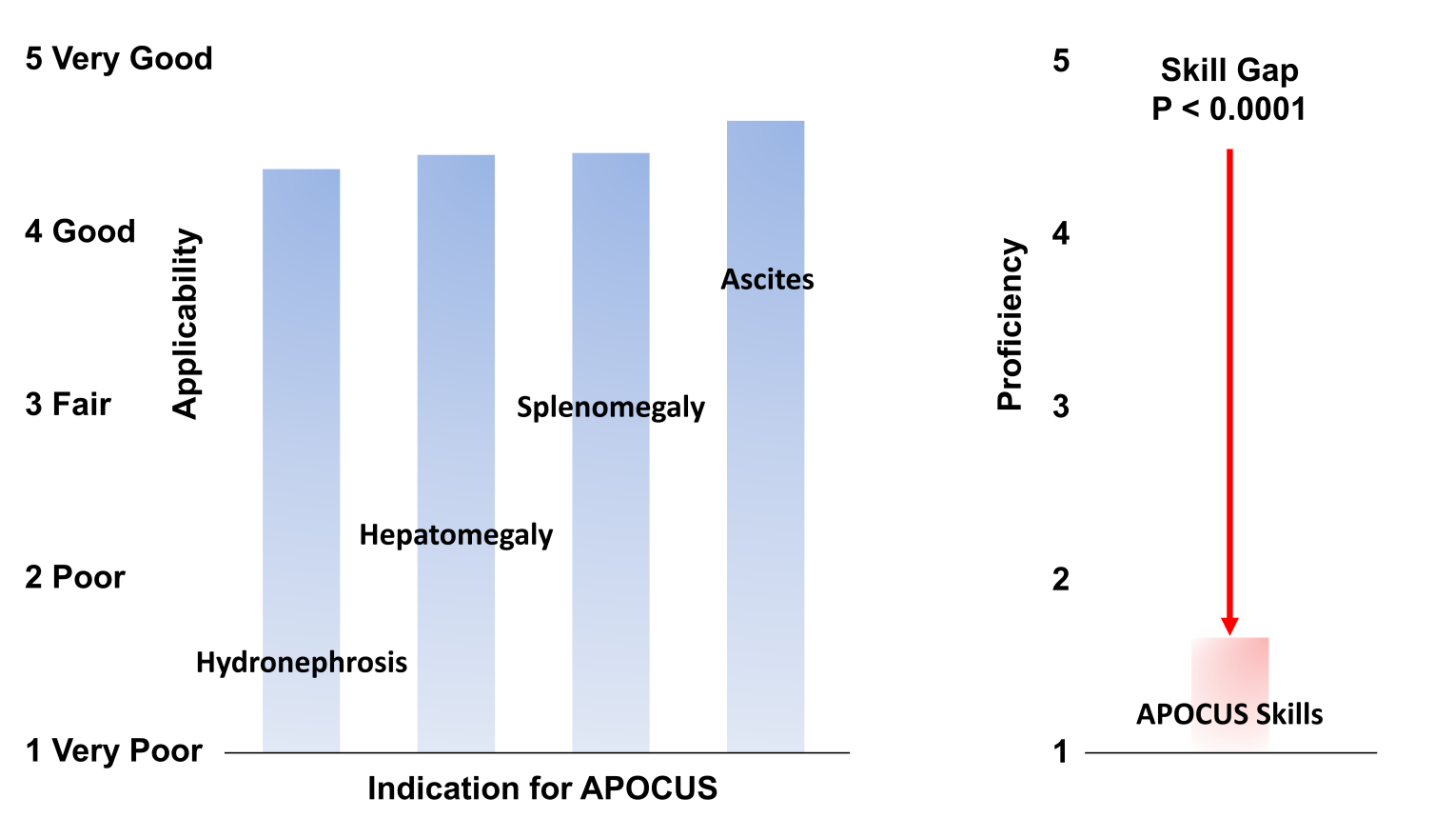
Residents’ perception of the applicability of APOCUS, self-reported proficiency, and the skill gap. This figure illustrates residents’ perceptions of the applicability of abdominal point-of-care ultrasound (APOCUS) to their clinical practice and their self-reported ability to perform APOCUS. Data are presented as mean ± standard deviation. The skill gap was calculated from the difference between the average applicability of all four indications for APOCUS and the mean self-reported proficiency in APOCUS.

The IM residents reported that APOCUS is very applicable to their practice (Figure 1 and Tables 2 and 3). All indications for APOCUS were thought to be highly applicable to IM practice, but scanning for abdominal free fluid was perceived to be the most applicable. However, the IM residents self-reported that their ability to perform APOCUS was poor. The assessment of the skill gaps can guide educational interventions to resolve this discrepancy.

### Evaluation of the skill gaps

The difference between self-reported ability to perform a skill and the perceived usefulness of that skill can be used to measure a skill gap 9. The applicability of APOCUS for detection of abdominal free fluid was reported as either good or very good by 93% of the sample. However, the sample’s self-reported ability to perform APOCUS (mean 1.66 ± SD 1.11; Figure 1 and Table 2) was significantly lower than their overall opinion of the least applicable indication for the use of APOCUS (mean 4.33 ± SD 1.04, P < 0.00001; Figure 1 and Table 2). These observations suggest the presence of significant skill gaps in APOCUS. This can only be addressed by institution of a training program.

We expected that the skills gap of junior residents (PGY1 and PGY2) would be greater than that of senior residents (PGY3 and PGY4). However, our data suggest there was no difference in their ability to perform APOCUS (Table 2). This may be because POCUS is a relatively new technology and very few trainers are available. Regardless, these observations reinforce the need for a POCUS training program. 

### Relevance of existing APOCUS training programs to Saudi Arabia

Perceptions of the applicability of APOCUS and the skill gaps reported by Canadian IM residency programs^9^ are similar to our observations in Saudi Arabia (Figure 1 and Table 2). This may be because APOCUS findings, whilst useful, are relatively non-specific. Thus, although there are regional differences in the epidemiology of intra-abdominal pathology^16^ and the availability of radiology services; the use of APOCUS to rapidly detect ascites at the bedside is universally applicable to the practice of medicine worldwide. This observation suggests that the international standardisation of basic APOCUS training may be possible and curricula developed in other countries may be relevant to internists in Saudi Arabia. 

### Strengths and Limitations

Whilst our study was conducted in IM residents, our observations and recommendations are likely to be relevant to nephrology, gastroenterology and hepatology fellows starting their fellowships. This is because the study was conducted towards the end of the academic year when the participating PGY4 IM residents had completed their residency training. 

Whilst the response rate to the survey was very high, the study has some limitations. Our data include self-reported knowledge. There are many potential causes of bias in such data.^20^ However, the ability to perform APOCUS was generally reported to be poor (Figure 1 and Tables 2 and 3). This finding is consistent with our personal observations.

Our study was conducted at only one institution in Riyadh, Saudi Arabia. So, its generalizability may be limited. However, our institution hosts one of the largest IM residency programs in Saudi Arabia. Our participants’ views are therefore likely to represent residents training in IM throughout Saudi Arabia and indeed other countries with well-developed healthcare systems. Our observations and their views on APOCUS should therefore be taken into account when developing training programs to safely and effectively integrate APOCUS into the practice of IM.

### Contribution to the existing literature

The presented data provide robust evidence that APOCUS is applicable to the practice of medicine in Saudi Arabia. However, our residents’ ability to perform APOCUS is poor. This is likely to be true throughout Saudi Arabia. So, our data suggest that Saudi Arabian IM residents have statistically and clinically significant skill gaps in APOCUS. 

Residency training programs must aim to provide tuition on the most clinically relevant knowledge and skills. Thus, IM residents clearly require a curriculum for training in APOCUS. Fellows in nephrology, gastroenterology and hepatology are also likely to benefit from formal training in APOCUS. Our observations can guide the development of a program that satisfies residents’ and fellows’ perceived needs. 

## Conclusions

Our data suggest that APOCUS is highly applicable to the practice of IM in Saudi Arabia. Existing programs for APOCUS training may be relevant to practice within the Middle East. Thus, international standardisation of APOCUS training may be possible and should be considered. Our findings will be of interest to those developing curricula to train residents and fellows in APOCUS.

## Sources of funding

This research did not receive any specific grant from funding agencies in the public, commercial, or not-for-profit sectors

## Conflicts of Interest

none declared.

## Supplementary Material 

Appendix 1
