# Supplementary material for: Measurement of the Applicability of Abdominal Point-of-Care Ultrasound to the Practice of Medicine in Saudi Arabia and the Current Skill Gaps
Source: POCUS J. 2021 Apr 22;6(1):36–41. doi: 10.24908/pocus.v6i1.14761 (PMC9979944; doi:10.24908/pocus.v6i1.14761)
Supplement: Appendix 1 [file pocusj-06-14761-s001.pdf]

Study of the applicability of abdominal point-of-care ultrasound (APOCUS) to medical practice in Saudi Arabia

Survey of residents' training, experience, accreditation, and knowledge of APOCUS

DEMOGRAPHIC

What specialty are you in?

Internal medicine

What stage of your career are you in?

Resident PGY-1 PGY-2 PGY-3 PGY-4

Gender Male Female

ULTRASOUND EXPERIENCE AND TRAINING

Did you receive / are you receiving formal training in APOCUS during post-graduate / residency training

No YES Approximate Total Number of Days of training

Do you have any formal accreditation in APOCUS?

No YES If Yes - what

How often do you use APOCUS in clinical practice?

Never

Once per month Once per week 3-4x per week Daily > Once/day

Please answer the following questions on a 5 point scale

1. Very poor 2. Poor 3. Fair 4. Good 5. Very Good

How applicable to the care of your patients are these indications for APOCUS?

Identifying hepatomegaly	1	2	3	4	5
Identifying splenomegaly	1	2	3	4	5
Identifying ascites/free fluid	1	2	3	4	5
Identifying hydronephrosis	1	2	3	4	5

Rate your current level of knowledge and skills in the following domain?

Knowledge of APOCUS and ability to interpret APOCUS findings (i.e. what is your level of proficiency in APOCUS)?

1. Very poor 2. Poor 3. Fair 4. Good 5. Very Good

Do you have any other comments or concerns?
